# Bibliometric analysis of traditional Chinese exercises in stroke rehabilitation from 2003 to 2022 using CiteSpace

**DOI:** 10.3389/fneur.2024.1260643

**Published:** 2024-02-01

**Authors:** Xiaoyi Li, Zhi Li, Hongxing Wang

**Affiliations:** Department of Rehabilitation Medicine, Zhongda Hospital Southeast University, School of Medicine, Southeast University, Nanjing, China

**Keywords:** stroke, traditional Chinese exercises, CiteSpace, visual analysis, bibliometric analysis

## Abstract

**Background:**

A comprehensive analysis was conducted on the relevant literature pertaining to the application of traditional Chinese exercises in stroke rehabilitation over the past 20 years. Additionally, a scientific knowledge map was created to elucidate the current research status, investigate its development process and research trends, and offer novel research perspectives for future studies.

**Methods:**

The data is sourced from the WOS Core Collection, and CiteSpace software is used to analyze the relevant literature on traditional Chinese exercises in stroke rehabilitation. The analysis began with the selection of publications, countries, institutions, highly cited authors, and co-cited references to summarize the current research status of traditional exercises in stroke rehabilitation. Second, keywords were employed to identify research hotspots, and keyword clustering time zone diagrams were chosen to track the research development process. Finally, burst keywords were employed to explore the research frontiers and trends in this field.

**Results:**

In total, 937 documents were retrieved, and the annual publication volume consistently and sustainably increased. China and the USA emerged as significant contributors. The Chinese University of Hong Kong had the highest publication count, with ADA L from the University of Sydney being a highly cited author. Initially, keywords focused on cardiac output, blood flow, pressure, and performance. Over time, the focus shifted to heart failure, muscle strength, mortality, and exercise capacity. Current trends encompass outcome, impact, virtual reality, and anxiety.

**Conclusion:**

Integrating key elements of traditional exercise approaches with the specific attributes of movement disorders during the stroke recovery phase is essential. Therefore, enhancing the stroke rehabilitation training program and exploring novel avenues for traditional exercise-based interventions are critical.

## 1 Introduction

Stroke remains the second leading cause of mortality and disability worldwide (1). Post-stroke dysfunction significantly affects patients’ physical functioning and quality of life, creating a substantial burden on families and society (2). Evidence-based medicine recognizes rehabilitation as an effective method for reducing stroke-related disabilities. Tailored rehabilitation interventions can improve post-stroke dysfunction and accelerate recovery (3).

Traditional Chinese exercises have a rich history spanning over 5,000 years and are classified as low- to medium-intensity aerobic exercises within the realm of modern medicine (4). Key traditional Chinese exercises encompass Taijiquan, Baduanjin, Yijinjing, Wuqinxi, Liuzijue, and Qigong. These exercises integrate posture control, breathing techniques, and mindfulness exercises to enhance meridian flow and blood circulation, thereby contributing to disease prevention and fostering physical and mental well-being (5). Due to their efficacy, affordability, ease of acquisition, and equipment-free nature, traditional Chinese exercises have garnered increasing attention within the clinical rehabilitation domain (6). In recent years, numerous researchers have investigated traditional Chinese exercises in stroke rehabilitation. However, a dearth of comprehensive analysis and organization within this research domain persists. This study examines the research findings, future research trends, and emerging areas of interest in this field and also furnishes references and recommendations for future investigations into traditional Chinese exercises for stroke rehabilitation.

## 2 Methods

### 2.1 Data source and collection

**Figure 1:**
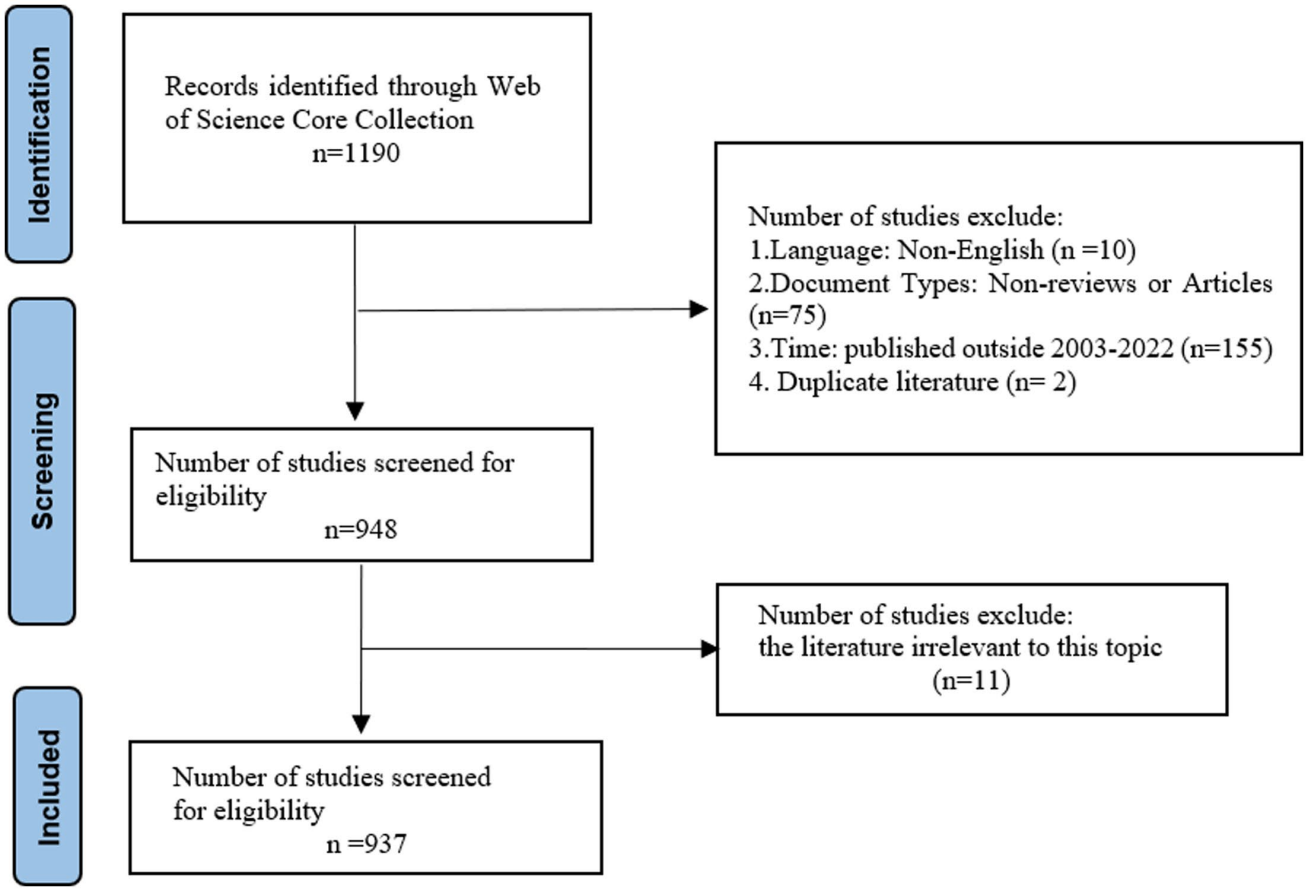
A flow diagram of study selection.

The data for the bibliometric analysis were sourced from the core collection of Web of Science (WOS). The search query includes the following terms: (stroke OR ischemic stroke OR apoplexy OR cerebrovascular disease OR cerebrovascular accident OR hemorrhagic stroke) AND (Tai-ji OR Tai Chi OR Chi, Tai OR Tai Ji Quan OR Ji Quan, Tai OR Quan, Tai Ji OR Taiji OR Taijiquan OR Taichi OR Tai-yi OR Tai Chi Chuan OR qigong OR qi gong OR chi gong OR breathing exercise OR ch’i Kung OR baduanjin OR ba duan jin OR Eight Brocade exercise OR eight section brocades OR Eight-sectioned Exercise OR wuqinxi OR five animal exercises OR yijinjing OR yi jinjing OR liuzijue OR traditional exercise OR Chinese traditional exercise OR traditional Chinese exercise OR Chinese exercise OR mind–body exercise). The language used for the search was English. The retrieval period spanned from 01 January 2003 to 31 December 2022. The document type selected was article and review. A total of 937 documents were retrieved in the search (Figure 1). Upon completion of the retrieval process, the data were saved as complete records and cited references. It was then exported in plain text format, renamed following the “download-XXX” convention, and imported into CiteSpace software (version 6.1.R6) for analysis. The software conducted a plagiarism check, and no duplicate documents were identified.

### 2.2 CiteSpace analysis methods

In our research, we applied CiteSpace for a comprehensive scientometric analysis of literature on traditional Chinese exercises in stroke rehabilitation, covering the period from 2003 to 2022. The slice length is set to 1, which means dividing the literature data into zones of “1 year” each; the source of subject terms and key paths are set to system defaults; Node Type includes countries, institutions, highly cited authors, co-cited references, keywords, and burst keywords for network analysis; depending on the analysis node, the threshold values Top N (referring to the number of items extracted in each time slice) and Top N% (referring to the top N% of items extracted in each time slice) are adjusted, and different pruning methods are selected.

### 2.3 Research methods

In our research, we utilized CiteSpace software, which integrates scientometrics, data and information visualization, and data mining algorithms. This article generates visualized scientific knowledge graphs that extract key information and research status from extensive literature data. These graphs facilitate the analysis of past and present research hotspots and development history as well as the exploration of active research frontiers and trends (7). The size of each node on the map indicates the frequency of the corresponding research object. Larger nodes represent a significant number of publications related to traditional Chinese exercises in stroke rehabilitation. The connections between nodes depict co-occurrence or co-citation relationships. The thickness of the lines represents the strength of the relationship, while the density of the graph connections reflects the degree of interconnectedness among the research objects (8). In this study, we initially analyze the quantity of literature, countries, institutions, highly cited authors, and co-cited references for analysis to summarize the current research status of traditional Chinese exercises in stroke rehabilitation. Subsequently, we employ keywords to identify research hotspots and employ keyword clustering time zone diagrams to track the research development process. Finally, we explore research frontiers and trends in this field by examining burst keywords.

## 3 Results

### 3.1 Literature volume analysis

**Figure 2:**
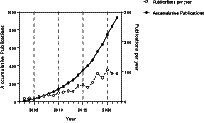
Annual and cumulative distribution of the trend map of publications.

The publication trend of literature volume reflects the change curve and time sequence of the number of publications on traditional Chinese exercises in stroke rehabilitation as a whole. As depicted in Figure 2, the annual publication volume of traditional Chinese exercises in the field of stroke rehabilitation research remained consistently low, always below 20, from 2003 to 2007. Starting from 2008, the number of relevant publications experienced a significant increase. Despite occasional declines in individual years, the overall growth trend of this research field can be observed from the annual publication volume in Figure 2, showing fluctuations. The cumulative number of published articles, as illustrated in Figure 2, indicates a relatively stable growth rate in this research field, suggesting that it will continue to exhibit a continuous growth trend in the coming years.

### 3.2 Country analysis

**Figure 3:**
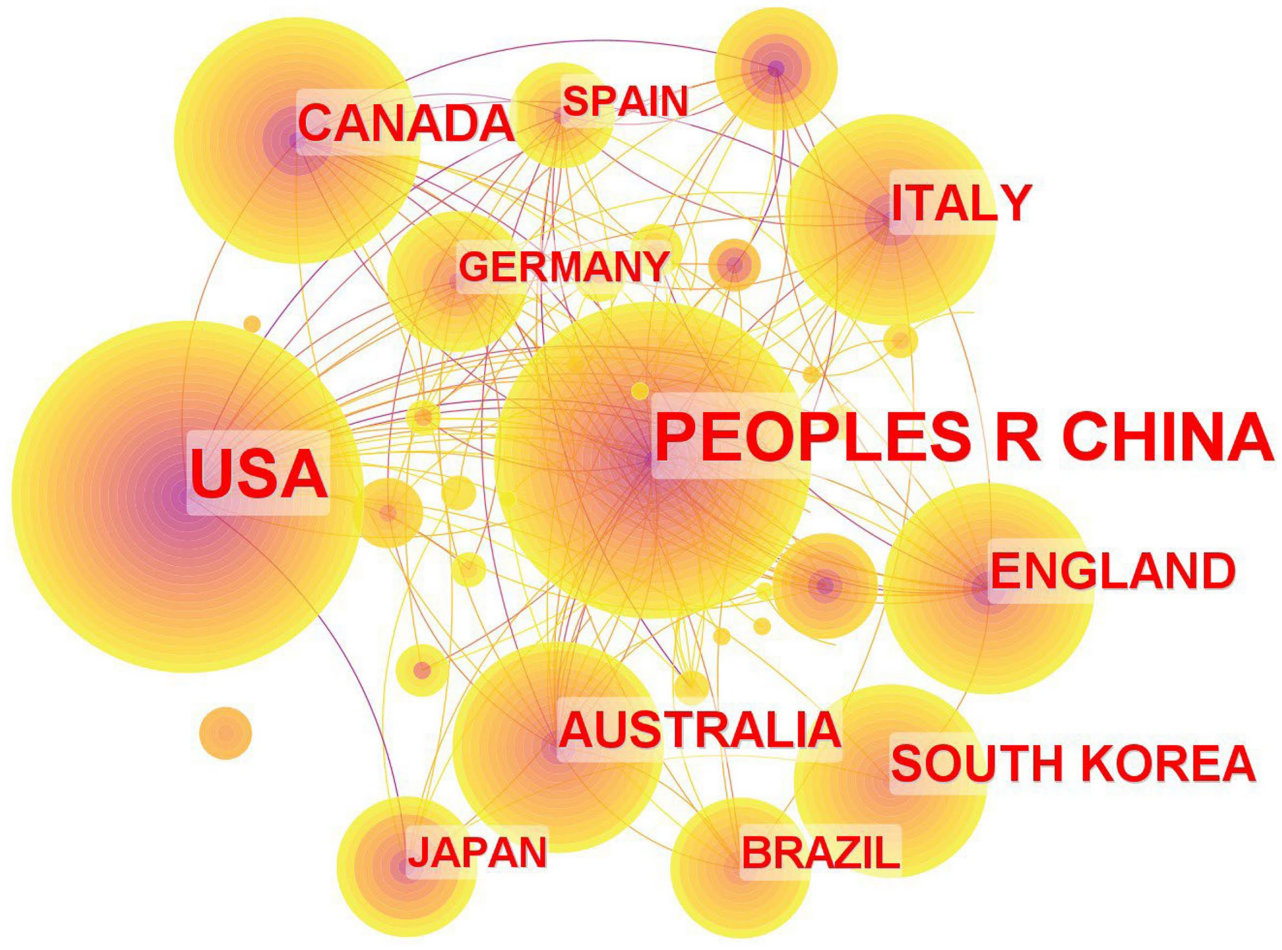
A country co-occurrence analysis map.

The countries are selected as nodes for analysis, and the country co-occurrence analysis map is generated as shown in Figure 3. Analyzing the distribution of literature publications by country provides valuable insights into countries with a significant number of publications on traditional Chinese exercises in stroke rehabilitation research, facilitating cross-country learning and communication.

As indicated in Table 1, China possesses the largest node with 262 published articles, securing the top position and accounting for 27.9% of the total publications, surpassing other countries by a significant margin. The United States takes the second position with 243 articles, representing 25.9% of the total published articles. Canada (64 articles, 6.8%) and Britain (54 articles, 5.7%) secured the third and fourth rankings, respectively. The United States holds the highest centrality of 0.80, signifying its extensive collaboration and exchanges with other countries and its pivotal role in international cooperation and exchanges. Following the USA, Australia, Singapore, and China sequentially rank second, third, and fourth in centrality, respectively. These countries have made significant contributions to fostering international cooperation and exchanges. Despite ranking fourth in centrality among all countries, China emerges as the frontrunner in terms of the number of published articles, suggesting its substantial research output in this field. However, future collaborations and exchanges with other countries need further strengthening.

**Table 1:** Top 8 countries in terms of frequency and centrality.

Country	Frequency	Country	Centrality
CHINA	262	USA	0.80
USA	243	AUSTRALIA	0.21
CANADA	64	SINGAPORE	0.21
ENGLAND	54	CHINA	0.18
AUSTRALIA	54	ENGLAND	0.16
ITALY	54	SAUDI ARABIA	0.16
SOUTH KOREA	49	CANADA	0.15
BRAZIL	32	SPAIN	0.14

### 3.3 Institutional analysis

**Figure 4:**
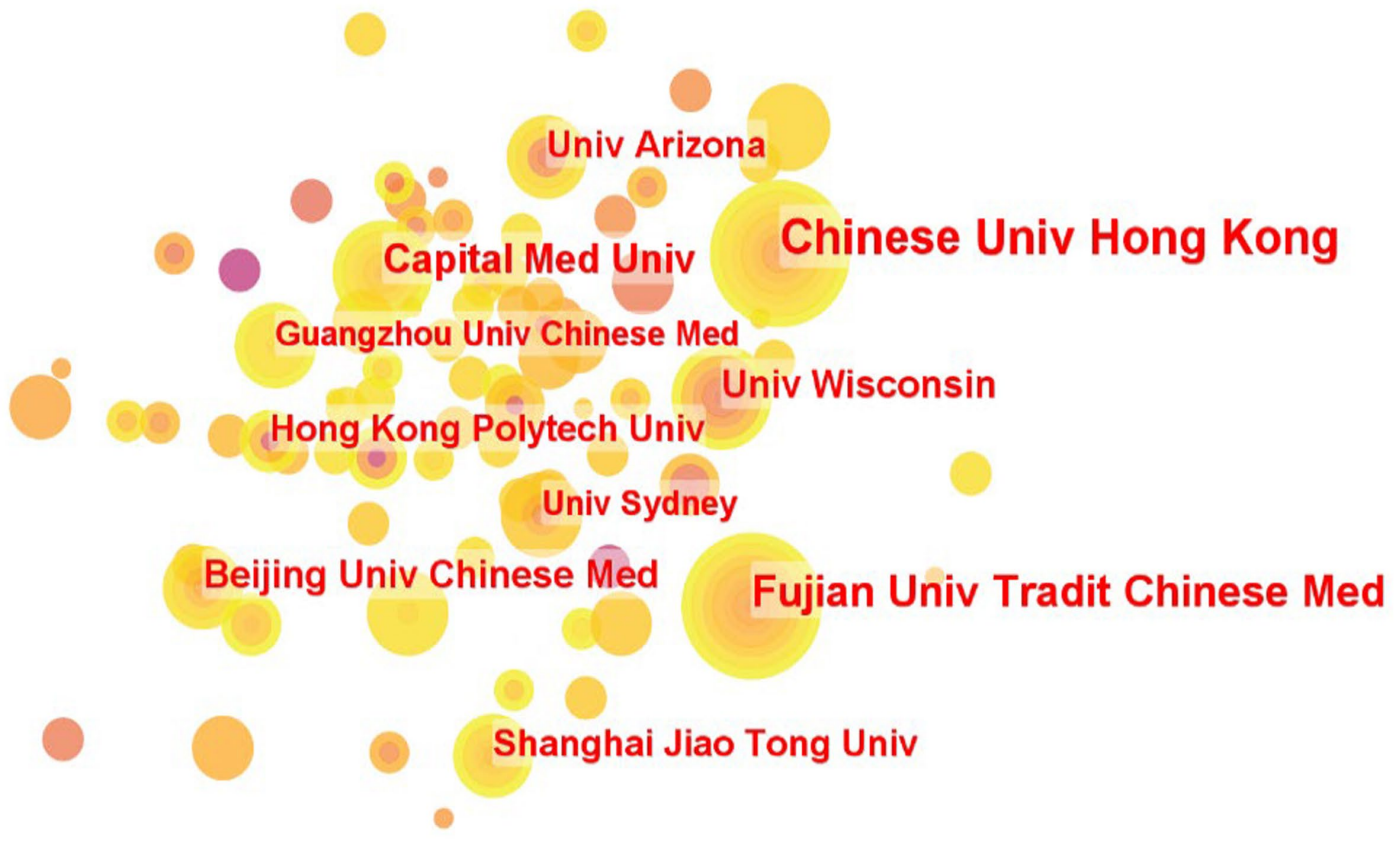
An institutional co-occurrence analysis map.

In our analysis, institutions are represented as nodes, resulting in the generation of an institutional co-occurrence analysis map, as shown in Figure 4. The number of publications and centrality of an institution serve as indicators of the institution’s research contribution and influence in this field. Analyzing institutions with significant publication volume and centrality facilitates the analysis of research topics and directions in this field.

As indicated in Table 2, the overall number of publications and centrality of these institutions are relatively low, suggesting that there is significant untapped research potential in this field and that various institutions can pursue further research. The Chinese University of Hong Kong occupies the top position as the institution with the highest number of publications, followed by the Fujian University of Traditional Chinese Medicine and the Capital Medicine University. These institutions demonstrate substantial research capacity in this field. The Chinese University Hong Kong exhibits the highest centrality, followed by the Shanghai Jiao Tong University and the Capital Medicine University, indicating that these institutions maintain extensive and close communication with other research institutions.

**Table 2:** Top 8 institutions in terms of frequency and centrality.

Institutions	Frequency	Institutions	Centrality
The Chinese University Hong Kong	26	The Chinese University Hong Kong	0.04
The Fujian University of Traditional Chinese Medicine	19	The Shanghai Jiao tong University	0.03
The Capital Medicine University	14	The Capital Medicine University	0.02
The Beijing University of Chinese Medicine	12	The Hong Kong Polytechnic University	0.01
The University of Wisconsin	12	The Guangzhou University of Chinese Medicine	0.01
The University of Arizona	11	The Shanghai University of Sport	0.01
The Shanghai Jiao Tong University	10	The Harvard University	0.01
The Hong Kong Polytechnic University	10	The University of Pittsburgh	0.01

### 3.4 Analysis of highly cited authors

**Figure 5:**
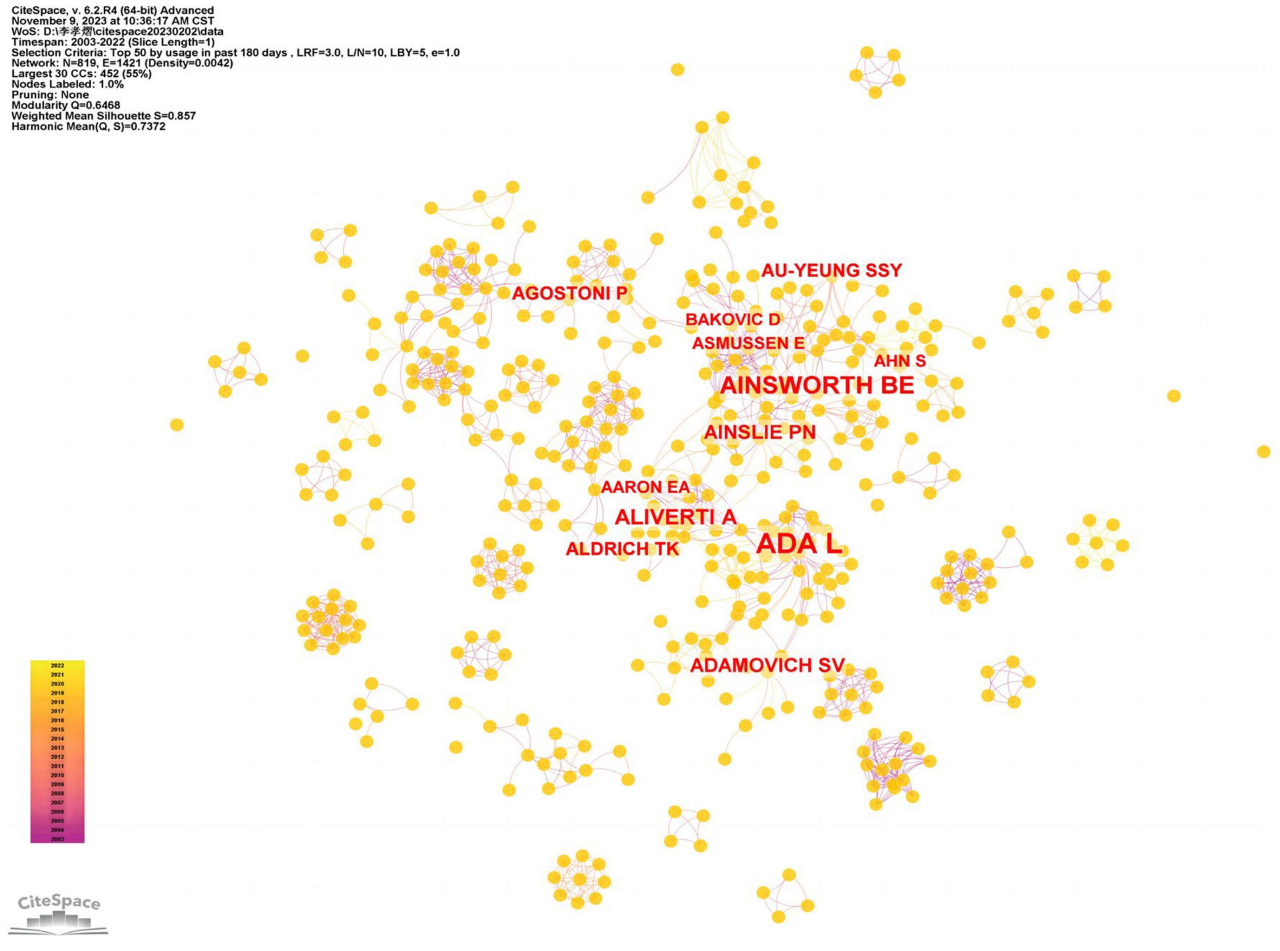
A co-citation analysis map of highly cited authors.

Choosing authors as the nodes for analysis, we obtain the co-citation analysis map of highly cited authors, as shown in Figure 5. Authors with high publication volume or citation rates can be considered core authors in the field, reflecting their academic influence and contributions to their research area. Analyzing the research themes and directions of core authors is beneficial for enhancing understanding of the field.

As shown in Figure 5, the network connections among various authors are dispersed around the core authors’ clustered network. The co-authorship network centered around ADA L from the University of Sydney is relatively dense, followed by networks centered around core authors, such as AINSWORTH BE from Arizona State University and ALIVERTI A from Politecnico di Milano. These core authors have close internal connections, but there is less communication and cooperation between different institutions. As shown in Table 3, in terms of publication volume, ADA L ranks first, indicating a high academic influence in the research field. Although AINSWORTH BE ranks second in publication volume, this core author’s centrality is the highest, indicating its essential role in communication and connection among scholars in the same field.

**Table 3:** Statistics on the frequency and centrality of highly cited authors.

Cited Authors	Institution	Country	Frequency	Cited Authors	Institution	Country	Centrality
ADA L	The University of Sydney	Australia	21	AINSWORTH BE	The Arizona State University	USA	0.08
AINSWORTH BE	The Arizona State University	USA	13	ANDERSEN LB	The Western Norway University of Applied Sciences	Norway	0.05
ALIVERTIA	Politecnico di Milano	Italy	9	ADA L	The University of Sydney	Australia	0.04
ADAMOVICH SV	The New Jersey Institute of Technology	USA	7	ASMUSSEN E	The University of Copenhagen	Denmark	0.03
AINSLIE PN	The University of British Columbia	Canada	7	ADAMS RP	The Princeton University	USA	0.02
ALDRICH TK	The Albert Einstein College of Medicine	USA	6	ADAMOVICH SV	The New Jersey Institute of Technology	USA	0.01
AU-YEUNG SSY	The Hong Kong Polytechnic University	China	6	AINSLIE PN	The University of British Columbia	Canada	0.01
AGOSTONI P	The University of Milan	Italy	6	AHN S	The University of British Columbia	Canada	0.01

### 3.5 Co-cited reference analysis

**Figure 6:**
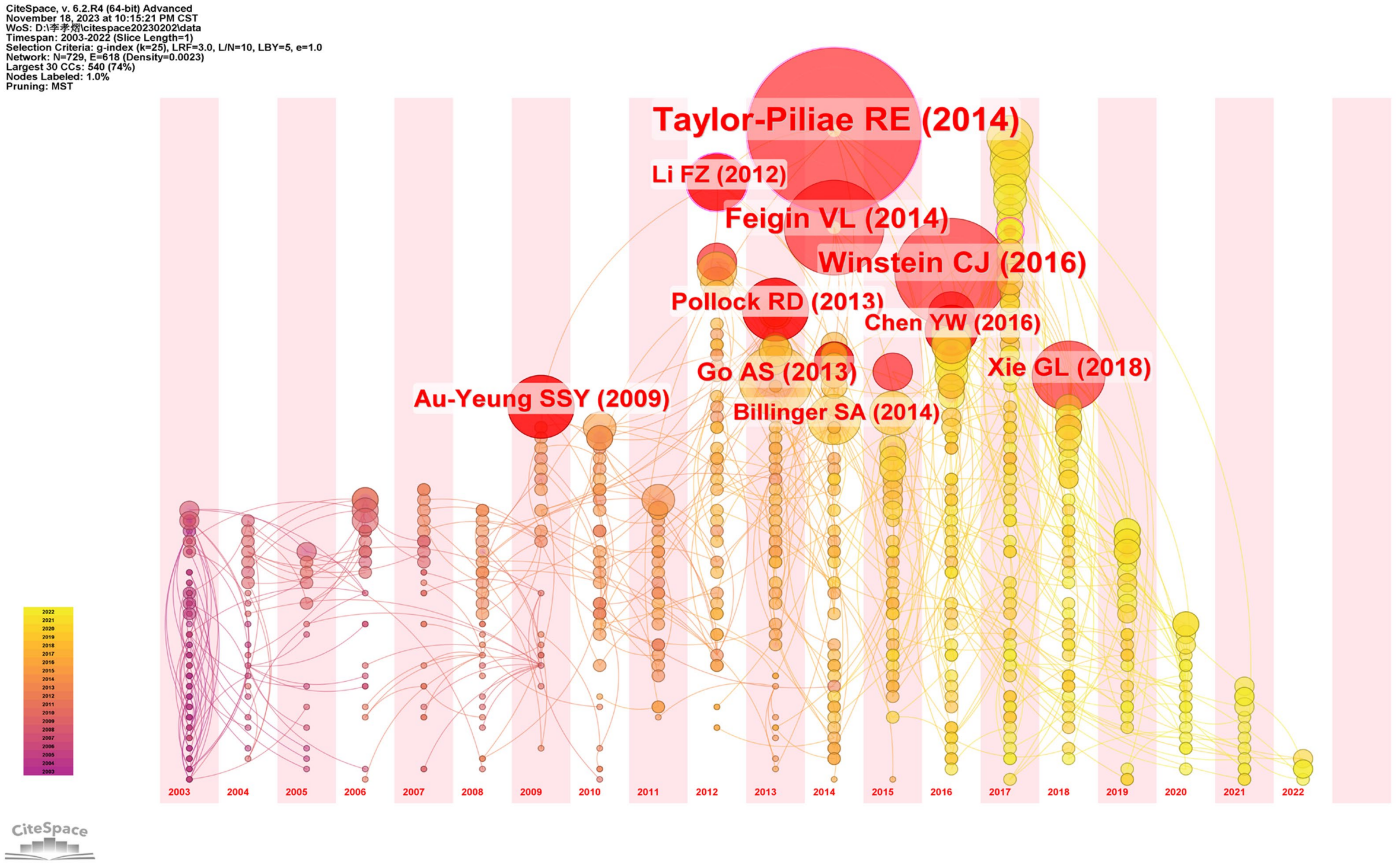
A co-cited reference timeline diagram.

The temporal distribution of references in the Co-Cited Reference Timeline Diagram allows us to identify dynamic trends and references of significant influence during different periods, shedding light on the field’s development and potential paradigm shifts. Choosing references as nodes for analysis, we obtain the co-cited reference analysis map shown in Figure 6. Each node represents a co-cited reference, with the nodes and connecting lines representing the relationships between references in the field and their co-citations. The larger the node, the more times that reference has been cited.

As Table 4 illustrates, the combined citations of these works exceed 100. This body of literature, encompassing both original research articles and reviews, establishes a solid theoretical groundwork for further investigation into traditional Chinese exercises and stroke rehabilitation. The first of these highly cited references is an article by Taylor-Piliae et al. (9) in ARCH PHYS MED REHAB. This study reveals that 12-week Tai Chi training enhances aerobic endurance and lowers fall rates among community-dwelling stroke patients, making it an ideal community-based exercise program. Following the article by Taylor-Piliae RE et al., the second most cited reference by Winstein et al. (10), published in STROKE, outlines adult stroke rehabilitation and care guidelines, highlighting the importance of teamwork and coordination in effective stroke recovery strategies. The third cited reference, authored by Feigin et al. (11) in LANCET, leverages data from the Global Burden of Diseases, Injuries, and Risk Factors Study 2010 to assess the global and regional impact of stroke from 1990 to 2010. Notably, while stroke mortality rates have declined over the past two decades, the overall global burden of stroke remains significant. Additionally, a pivotal study by Go et al. (12) in CIRCULATION, focusing on heart disease and stroke, highlights the ongoing challenge of managing and monitoring cardiovascular risk factors. A study published in STROKE underscores a general lack of physical activity among stroke survivors, pointing out that exercise regimens can substantially enhance their functional capabilities and quality of life while also mitigating the risk of cardiovascular events. This research advocates for patient engagement in low- to moderate-intensity aerobic activities, muscle-strengthening exercises, reduced sedentary behavior, and effective management of secondary stroke risk factors (13). Studies 4 (14) and 6 (15) illustrate how Tai Chi training can significantly improve balance in stroke patients, suggesting its suitability for community-based stroke programs. Tai Chi’s emphasis on precise joint positioning is known to heighten proprioceptive abilities. In another vital contribution, the eighth article (16) published in NEW ENGL J MED validates that Tai Chi training enhances balance in patients with mild-to-moderate Parkinson’s disease, thereby lowering their risk of falls. The seventh article (17), a comprehensive systematic review, draws attention to the impaired respiratory muscle strength in stroke patients, which increases their susceptibility to chest infections. The article advocates for respiratory training to bolster respiratory function. Finally, the tenth study (18) consolidates current research on the efficacy of Tai Chi in managing four prevalent chronic conditions: cancer, osteoarthritis, heart failure, and chronic obstructive pulmonary disease. This research confirms the beneficial impact of Tai Chi in enhancing physical functionality across these conditions.

**Table 4:** Top 10 most frequently cited references.

Rank	Frequency	Year	Journal	Impact factor	Journal ranking	Co-cited reference
1	26	2014	ARCH PHYS MED REHAB	4.3	Q1	Taylor-Piliae RE, 2014, ARCH PHYS MED REHAB, V95, P816, DOI: 10.1016/j.apmr.2014.01.001
2	17	2016	STROKE	8.3	Q1	Winstein CJ, 2016, STROKE, V47, PE98, DOI: 10.1161/STR.0000000000000098
3	15	2014	LANCET	168.9	Q1	Feigin VL, 2014, LANCET, V383, P245, DOI: 10.1016/S0140-6736(13)61953-4
4	11	2018	EUR REV AGING PHYS A	6.3	Q1	Xie GL, 2018, EUR REV AGING PHYS A, V15, P0, DOI: 10.1186/s11556-018-0206-x
5	11	2013	CIRCULATION	37.8	Q1	Go AS, 2013, CIRCULATION, V127, PE6, DOI: 10.1161/CIR.0b013e31828124ad
6	10	2009	NEUROREHAB NEURAL RE	4.2	Q1	Au-Yeung SSY, 2009, NEUROREHAB NEURAL RE, V23, P515, DOI: 10.1177/1545968308326425
7	10	2013	INT J STROKE	6.7	Q1	Pollock RD, 2013, INT J STROKE, V8, P124, DOI: 10.1111/j.1747-4949.2012.00811.x
8	9	2012	NEW ENGL J MED	158.5	Q1	Li FZ, 2012, NEW ENGL J MED, V366, P511, DOI: 10.1056/NEJMoa1107911
9	8	2014	STROKE	8.3	Q1	Billinger SA, 2014, STROKE, V45, P2532, DOI: 10.1161/STR.0000000000000022
10	8	2016	BRIT J SPORT MED	18.4	Q1	Chen YW, 2016, BRIT J SPORT MED, V50, P397, DOI: 10.1136/bjsports-2014-094388

### 3.6 Analysis of research hotspots based on keyword co-occurrence

**Figure 7:**
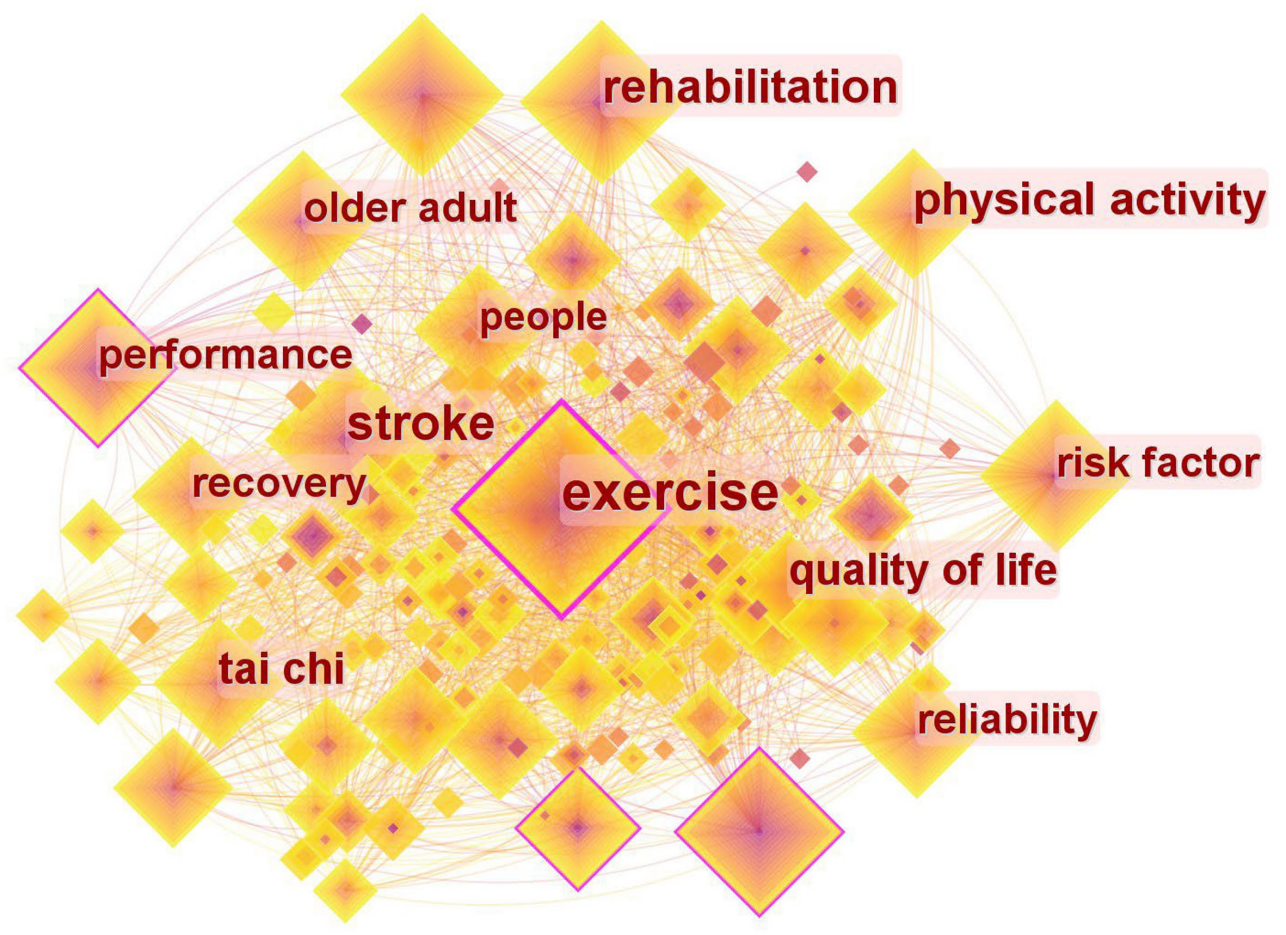
A map of the analysis of keywords.

Keywords are selected as nodes to generate the keyword analysis map depicted in Figure 7. Keywords represent the extracted and summarized core viewpoints and key content of the literature. Analyzing keywords with high frequency and centrality within specific periods allows for the identification of research topics and hotspots in this field.

Table 5 provides a statistical ranking of the frequency and centrality of each keyword. The keyword “exercise” exhibits the highest frequency and centrality, indicating the research focus on exercise for stroke patients. Studies have demonstrated that exercise improves physical function, psychological well-being, and quality of life in stroke patients (19). Brain function plasticity serves as the foundation for limb function recovery in stroke patients (20), while physical activity fosters the process of brain function recovery in stroke patients (21). Traditional Chinese exercises, a form of physical activity, highlight active patient participation and attention concentration, facilitating more effective rehabilitation outcomes

**Table 5:** Top 10 keywords in terms of frequency and centrality.

Keywords	Frequency	Keywords	Centrality
Exercise	252	Exercise	0.36
Stroke	146	Performance	0.15
Rehabilitation	111	Cardiac output	0.11
Physical activity	95	Blood pressure	0.10
Tai chi	83	Cardiovascular disease	0.10
Quality of life	79	Stroke	0.08
Older adult	67	Rehabilitation	0.07
Performance	66	Risk factor	0.07
Risk factor	64	Ischemic stroke	0.07
Recovery	62	Health	0.06

### 3.7 Research evolution analysis based on keyword clustering co-occurrence time zone map

**Figure 8:**
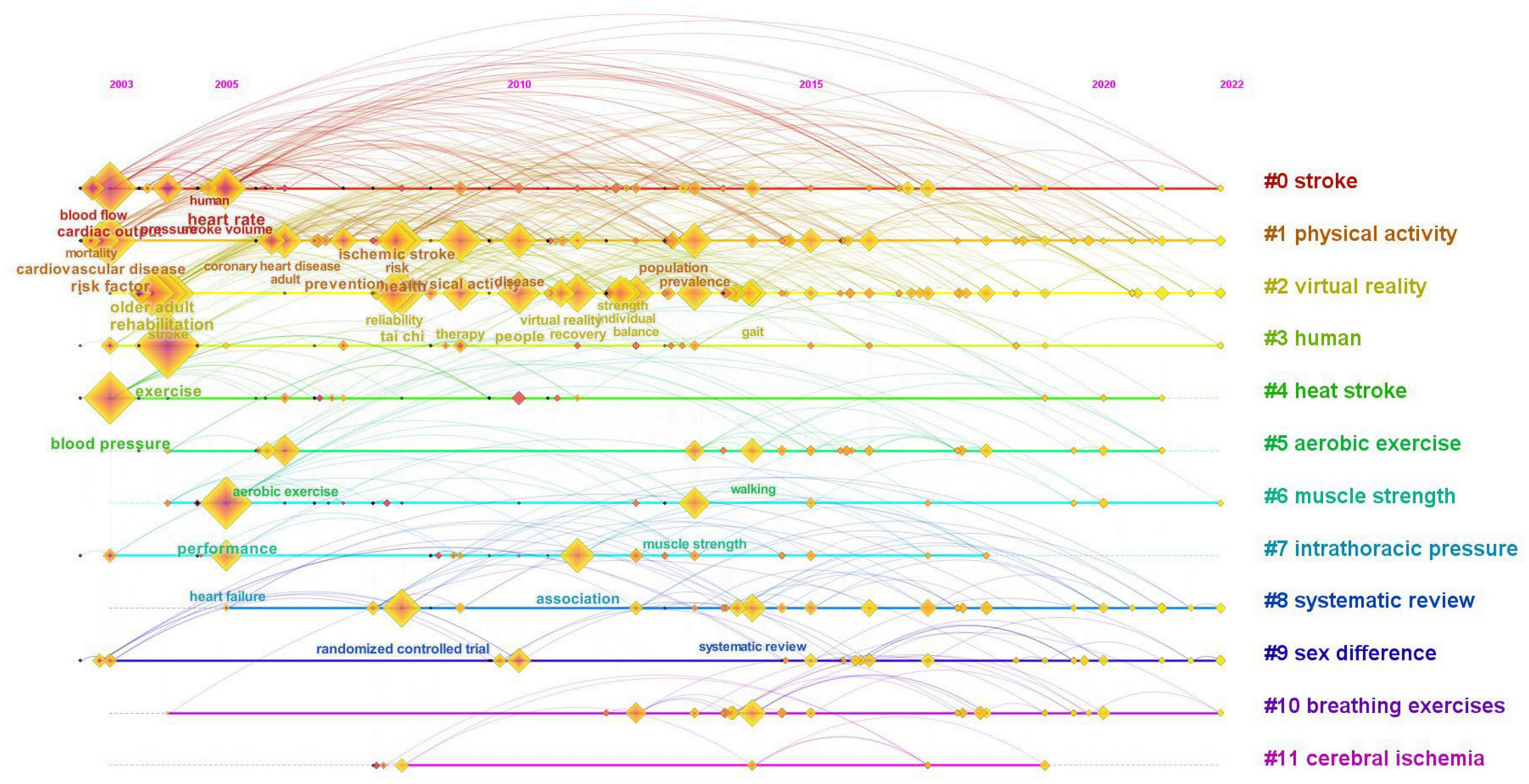
A keyword cluster co-occurrence time zone map.

We conducted a cluster analysis on the literature keywords. In addition, we used the year of keyword appearance and cluster labels as the X and Y axes of the map to generate the keyword cluster co-occurrence time zone map in Figure 8. The time zone map of keyword clustering co-occurrence illustrates the duration of keyword clustering and the co-occurrence relationships between keywords, providing insights into the development and evolution of the research field. The domains encompassing stroke (# 0), physical activity (# 1), virtual reality (# 2), aerobic exercise (# 5), and muscle strength (# 6) exhibited a significant duration, indicating the importance of these clusters in the field.

### 3.8 Analysis of research fronts based on burst keywords

**Figure 9:**
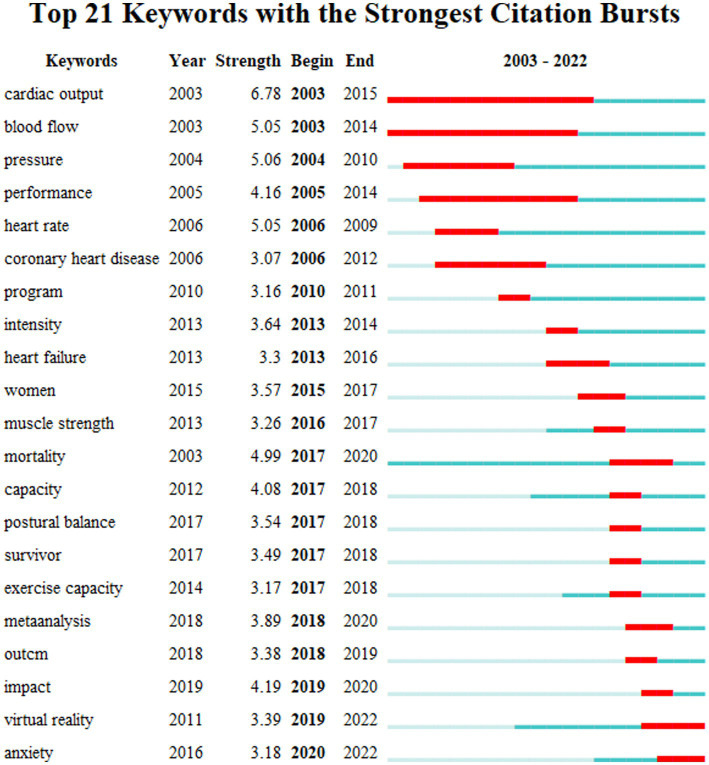
Top 21 keywords with the strongest citation bursts.

“Burst keywords” are terms characterized by a notably high-frequency change over a specific time frame. This concept is instrumental in identifying and tracking the evolution and forthcoming directions of a particular academic field. The analysis of burst keywords, as depicted in Figure 9, illustrates a dynamic shift in research focus. Between 2003 and 2012, the prevailing trends centered around topics, such as cardiac output, blood flow, pressure, performance, heart rate, and coronary heart disease. In the subsequent period from 2013 to 2018, there was a noticeable pivot toward areas such as heart failure, muscle strength, mortality, capability, postural balance, survival, and exercise capacity. In the current phase, terms such as outcome, impact, virtual reality, and anxiety are progressively becoming research hotspots and trends.

## 4 Discussion

### 4.1 Summary of findings

In the realm of stroke rehabilitation, traditional Chinese exercises are rapidly emerging as a significant therapeutic approach for functional disorders. This evolution is underpinned by a surge in research focused on its clinical utility, significantly enhancing the recognition of traditional Chinese exercises’ efficacy. A global analysis identifies China, the USA, Canada, and England as frontrunners in this discipline. Notably, institutions, such as the Chinese University of Hong Kong, the Fujian University of Traditional Chinese Medicine, and the Capital Medical University, are leading the charge, with the Chinese University of Hong Kong being particularly notable for its exemplary international collaborations. Author network assessments reveal a concentrated network with ADA L from the University of Sydney at its core, highlighting their influential role in this specialized field.

Research evolution and hotspot analysis in the field of traditional Chinese exercises have demonstrated substantial advancements, ranging from a fundamental grasp to broadening its applications and innovations in methodology. The study by Taylor-Piliae et al. (9) underscores Tai Chi’s significant impact on enhancing aerobic endurance and reducing patient fall incidents, thus validating traditional Chinese exercises as an effective community rehabilitation tool. Additionally, the stroke rehabilitation and care guidelines by Winstein et al. (10) emphasize teamwork’s pivotal role in rehabilitation, aligning with the integration of traditional Chinese exercises into contemporary rehabilitation strategies. These findings align with the results of keywords analysis and clustering co-occurrence time zone map, the latter of which reveals the significance of keywords such as physical activity, aerobic exercise, muscle strength, and virtual reality, indicating that research in these areas is expanding and becoming more comprehensive.

Burst keywords analysis reveals a shift in research focus within traditional Chinese exercises, moving from initial physiological and cardiovascular studies to more pragmatic aspects, such as muscle strength, exercise capacity, and long-term survival. This shift signifies a growing acknowledgment of the role of traditional Chinese exercises in stroke rehabilitation, extending beyond physical enhancement to improving overall life quality and mental health. Feigin et al. (11), utilizing global disease burden data, underscore the profound impact of stroke, thereby emphasizing the urgency for efficacious rehabilitation strategies. The research by Go et al. (12) highlights the necessity of managing cardiovascular risk factors in stroke rehabilitation, which is linked to lifestyle changes. This finding underscores the need for future research to concentrate on preventive strategies in rehabilitation, including intensified aerobic exercise and reducing sedentary lifestyles. Traditional Chinese exercises, emphasizing balance and functional recovery, present a holistic approach to stroke rehabilitation, blending traditional and contemporary techniques. Hence, future studies should aim to develop standardized traditional Chinese exercise rehabilitation protocols, enhancing patients’ physiological, psychological, and overall quality of life.

### 4.2 Hot issues of traditional Chinese exercises and stroke dysfunction rehabilitation

Aerobic exercise training enhances mobility, mental health, and overall quality of life for stroke patients. The American Heart Association advocates for routine aerobic exercise in both stroke prevention and treatment (10). Research has established that cerebrovascular hemodynamic disorders play a critical role in stroke’s pathophysiology (22). Aerobic activities can mitigate or even reverse arterial stiffness by fostering arterial remodeling, reducing sympathetic nervous activity, and enhancing cerebrovascular hemodynamics. A study by Zheng et al. (23) found that a 12-week Tai Chi program offers potential benefits for cerebrovascular hemodynamics in elderly individuals at risk of stroke. Moreover, peak oxygen uptake, a prime measure of aerobic capacity and a key predictor of mortality in cardiovascular disease, is approximately 20% higher in Tai Chi practitioners than in inactive individuals. Long-term Tai Chi practice is associated with improved cardiovascular health and a reduced risk of stroke (24).

Preventing stroke effectively involves managing modifiable risk factors such as hypertension, hyperlipidemia, hyperglycemia, lack of exercise, and obesity, which are more controllable compared to clinical treatment. Hypertension is the primary modifiable risk factor for stroke, with regular physical exercise being a key preventative and therapeutic strategy for hypertension (25). Research indicates that a 12-week Tai Chi training significantly lowers systolic and diastolic blood pressure, aiding in effective blood pressure management (26). Additionally, a long-term Tai Chi practice enhances bone density, serving as a cost-effective approach to osteoporosis prevention (27). By addressing stroke’s high-risk factors, traditional Chinese exercises play a vital role in its primary prevention.

Reintegration into society and family is a critical, long-term objective in stroke rehabilitation. However, this reintegration does not signify the end of rehabilitation needs, as ongoing treatment is often required. Continuing rehabilitation at home or in the community is, therefore, crucial. Traditional Chinese exercises, known for their ease of learning, moderate intensity, suitability for various individuals, and independence from economic and locational constraints, facilitate remote rehabilitation via telemedicine platforms. This approach enables innovative rehabilitation, allowing patients to continue their recovery in community and home settings. The significance of traditional Chinese exercises in remote rehabilitation was particularly evident during the COVID-19 pandemic, providing vital post-discharge treatment for stroke patients. A study by Tousignant et al. (28) validated the effectiveness of Tai Chi in home-based remote rehabilitation for stroke patients. Such remote rehabilitation ensures the continuity and feasibility of rehabilitation services, both at home and within the community. Furthermore, integrating remote rehabilitation with virtual reality can further enhance functional training for stroke patients.

#### 4.2.1 Traditional Chinese exercises and motor dysfunction in stroke

Balance and coordination dysfunctions are prevalent among stroke patients. Traditional Chinese exercises, characterized by their slow, relaxed, and rhythmic movements, are particularly beneficial for those with motor impairments. These exercises help to strengthen muscles, increase endurance, and improve motor skills and balance (29, 30).

Tai Chi, involving exercises such as shifting the center of gravity, trunk rotation, and single-leg standing in various positions, effectively enhances balance and coordination control in these patients (31). The Tai Chi gait, marked by lower impact forces and more balanced weight distribution between the front and rear foot, contributes to a reduced fall risk (32). Research by Tsang et al. (33) demonstrates that Tai Chi practitioners exhibit superior knee joint proprioception and standing balance control compared to controls. Furthermore, studies by Kwok et al. (34) suggest that Tai Chi training enhances hand-eye coordination and balance control, showing increased accuracy in both stationary and moving visual signal tasks. Addressing the risk of falls in subacute stroke patients, research by Zhao et al. (35) has adapted traditional Tai Chi into a seated form, minimizing fall risks while maintaining its rehabilitation efficacy for these patients.

Core muscle group training, commonly employed in clinical balance training, hinges significantly on the role of deep core muscles for trunk stabilization (36). These muscles include the diaphragm, transversus abdominis, and pelvic floor muscles, all of which are central to respiratory functions and trunk control (37). Effective core stability, crucial for correct postural control, is fundamentally linked to proper breathing techniques (38). The Liuzijue, a technique focusing on breath regulation through varying mouth shapes during inhalation and exhalation, facilitates intra-abdominal pressure control. This method activates core muscles and enhances trunk coordination (39). Research by Zhang et al. (40) and Wang et al. (41) indicates that the Liuzijue, integrating breathing-based exercises, is more effective in improving balance function in stroke patients through deep core muscle activation than traditional core stability training. The Baduanjin, involving symmetric postures and coordinated movements, requires patients to shift their center of gravity and control lower limb and trunk movements, thereby extending balance maintenance. Studies by Liao et al. (42) demonstrate that this practice enhances muscle strength, balance, and coordination on the hemiplegic side, aiding in the rehabilitation of affected limb functions. Additionally, the Baduanjin includes exercises targeting joint mobility and body ligaments (43).

#### 4.2.2 Traditional Chinese exercises and respiratory dysfunction in stroke

The Liuzijue technique presents distinct advantages in ameliorating post-stroke dysarthria. Stroke patients with dysarthria maintain basic language skills but struggle with muscle dysfunction affecting articulation, breathing, phonation, rhythm, and resonance (44). Recovery from post-stroke dysarthria heavily relies on improved breath control. Traditional breathing training methods, such as blowing out candles or paper, mainly emphasize the shift from thoracic to abdominal breathing, but they overlook the simultaneous management of pronunciation and breathing. These methods predominantly build muscle strength and offer a limited treatment scope (45). In contrast, combining the Liuzijue technique with basic phonation training significantly enhances breath control and overall speech capabilities, aiding patients in developing a normalized speech breathing pattern (46).

#### 4.2.3 Traditional Chinese exercises and cognitive dysfunction in stroke

Research shows that cognitive dysfunction is a common aftermath of stroke, predominantly manifesting as memory impairment, diminished concentration, and reduced executive and computational skills. This cognitive decline significantly hampers the daily living abilities and overall quality of life of stroke survivors (47). Yi Jin Jing, a traditional Chinese exercise, has been found to positively affect both the physical and mental health of these patients. A study by Xue et al. (48) indicates that Yi Jin Jing, when compared to standard rehabilitation therapies, is more effective in enhancing cognitive dysfunction following a stroke.

#### 4.2.4 Traditional Chinese exercises and mental and emotional dysfunction in stroke

Stroke survivors frequently encounter negative emotions, including anxiety, depression, and sorrow. Traditional Chinese exercises encourage a tranquil state of mind and body, emphasizing harmonious breathing for optimal physical and mental relaxation (49). This approach effectively eases sympathetic nervous system tension and enhances mood. Mental relaxation, achieved through these practices, is also associated with reduced fatigue (50). Extensive research confirms that regular Tai Chi practice contributes to better mental health, notably stress reduction, anxiety relief, depression mitigation, and the fostering of positive emotions (51, 52). The Baduanjin, integrating physical movements with mental regulation, has been found to improve neurohumoral regulation, bolster immunity, and enhance sleep quality, thereby promoting overall physical well-being in stroke survivors (53). Given that poor sleep quality is a common challenge for stroke patients (54), Tai Chi has been identified as a beneficial practice for improving sleep in individuals with insomnia, positioning it as a viable complementary or alternative therapeutic option (55).

### 4.3 Directions for improvement and challenges

The exploration of traditional Chinese exercise practices in stroke rehabilitation reveals significant opportunities for improvement and further research. A key challenge is the lack of standardized and specific operational guidelines for these methods, affecting their comparability across various studies and clinical settings. Additionally, the field is marked by a scarcity of robust clinical trial designs, particularly concerning long-term follow-up studies, which restricts a comprehensive understanding of the enduring effects of these practices. Given the diverse cultural backgrounds and personal preferences of patients, there is a pressing need for future research to focus more on developing personalized rehabilitation programs. Therefore, future efforts in this domain must be more meticulous and inclusive, aiming to improve the quality of research and effectively address the rehabilitation needs of stroke patients.

### 4.4 Limitations

First, our analysis exclusively considered pertinent literature from the Web of Science database, potentially constraining the scope of the research. Second, the study focused on articles and reviews, omitting conference records, letters, and books, which could introduce biases. Third, the study considered publications in English. However, in non-English-speaking regions, researchers might predominantly explore traditional Chinese exercises. This potential bias warrants attention. Finally, traditional Chinese exercises are profoundly shaped by traditional Chinese philosophical principles and wellness concepts. Diverse regions show varying degrees of acceptance influenced by their unique historical backgrounds and the influence of economic factors. Prosperous regions receive advantages from governmental or societal support, whereas economically disadvantaged areas encounter resource constraints. Furthermore, societal preferences significantly influence the adoption of these exercises. Some prioritize both physical and mental well-being, while others opt for alternative exercise forms and spiritual activities. In conclusion, the varying regional perspectives on the effectiveness of traditional Chinese exercises result from cultural, historical, and economic influences. Analyzing these distinctions can enhance our holistic comprehension of their adaptability, offering insights for promotional and preservation initiatives. Future research should further investigate regional disparities to offer a comprehensive view of the global landscape of traditional exercises within the context of stroke rehabilitation.

## 5 Conclusion

Considering the varying location and severity of stroke injuries, it is crucial to tailor and individualize the training of traditional Chinese exercises. In a study conducted by Li et al. (56), it was discovered that personalized therapeutic Tai Chi training was superior to conventional training methods in reducing the occurrence of falls among community-dwelling older adults at a high risk of falls. Moving forward, utilizing contemporary rehabilitation theory to optimize and enhance traditional Chinese exercises is essential. This entails integrating the technical aspects of traditional exercise with the specific features of movement disorders during stroke recovery. Moreover, there is a need to expand the stroke rehabilitation training program and explore innovative approaches to traditional exercise rehabilitation.

In summary, the utilization of traditional Chinese exercises in stroke rehabilitation demonstrates significant potential for advancement. In the future, researchers may prioritize reducing the risk of stroke and implementing effective primary prevention strategies. Despite notable advancements in this field, a review and analysis using CiteSpace software reveals ongoing challenges and obstacles in its future progress. Overcoming these hurdles necessitates sustained innovation and diligent efforts from researchers worldwide.

## Data availability statement

The original contributions presented in the study are included in the article/supplementary material, further inquiries can be directed to the corresponding author.

## Author contributions

HW: Conceptualization, Funding acquisition, Supervision, Writing – original draft, Writing – review & editing. XL: Formal analysis, Investigation, Methodology, Visualization, Writing – original draft. ZL: Data curation, Investigation, Writing – original draft.
